# Single-cell analysis defines the lineage plasticity of stem cells in cervix epithelium

**DOI:** 10.1186/s13619-021-00096-2

**Published:** 2021-11-01

**Authors:** Zixian Zhao, Yujia Wang, Yingchuan Wu, Dandan Li, Ting Zhang, Yu Ma, Xiaoming Teng, Wei Zuo

**Affiliations:** 1 East Hospital, School of Medicine, Tongji University, Shanghai, China; 2 Super Organ R&D Center, Regend Therapeutics, Shanghai, China; 3 Shanghai Jincai East Secondary School, Shanghai, China; 4 Shanghai First Maternity and Infant Hospital, Tongji University, Shanghai, China; 5 Ningxia Medical University, Yinchuan, China; 6 The First Affiliated Hospital, Guangzhou Medical University, Guangzhou, China

**Keywords:** Cervix, Stem cells, Regeneration, Single-cell RNA-seq

## Abstract

Information about the dynamic change and post-injury regeneration of cervical epithelium is relatively rare, even though it is tightly related to gynecologic malignancy. Here, using a feeder cell-based culturing system, we stably cloned mouse and human P63 and KRT5 expressing cells from the adult cervix as putative cervical stem/progenitor cells (CVSCs). When subjected to differentiation, the cultured cells gave rise to mature cervical epithelium by differentiating into squamous or glandular cells. The ability of endogenous mouse CVSCs to reconstitute cervical epithelium after injury was also evident from the genetic lineage tracing experiments. Single-cell transcriptomic analysis further classified the CVSCs into three subtypes and delineated their bi-lineage differentiation roadmap by pseudo-time analysis. We also tracked the real-time differentiation routes of two representing single CVSC lines in vitro and found that they recapitulated the predicted roadmap in pseudo-time analysis. Signaling pathways including Wnt, TGF-beta, Notch and EGFR were found to regulate the cervical epithelial hierarchy and implicated the different roles of distinct types of cells in tissue homeostasis and tumorigenesis. Collectively, the above data provide a cloning system to achieve stable in vitro culture of a bi-lineage stem/progenitor cell population in the cervix, which has profound implications for our understanding of the cervix stem/progenitor cell function in homeostasis, regeneration, and disease and could be helpful for developing stem cell-based therapies in future.

**Supplementary Information:**

The online version contains supplementary material available at 10.1186/s13619-021-00096-2.

## Background

During the lifetime, the uterine cervical epithelium undergoes several dramatic changes in histomorphology. These alterations start as early as fetal development and continue to the menopause, presenting a dynamic cervix epithelium (Singer 1977; Hwang et al. 2009). The most prominent change of this process is squamous metaplasia, which involves the gradual replacement of the endocervical columnar epithelium by a new squamous epithelium at the squamo-columnar junction that lines the cervical canal (Suh and Silverberg 1990). It can be observed before birth and is particularly prominent during adolescence (Reich et al. 2017). Universally, metaplasia is a precursor to low-grade dysplasia, which can culminate in high-grade dysplasia and carcinoma (van der Marel et al. 2014). The causes of metaplasia including hormone production, growth of vaginal bacterial flora, inflammation, and virus infection. It is also assumed that a stem/progenitor cell population may play a role in both squamous metaplasia and the potential carcinogenesis in the cervix since it is suggested to be the target cell for the human papillomavirus infection (Schiffman et al. 2007; Martens et al. 2004). Since cervical carcinoma can be of a squamous or a glandular type, the target cell in the carcinogenic cascade should also be able to differentiate into both lineages.

Histologically the squamous epithelium adjacent to the junctional interface is composed of a basal layer, an intermediate sub-columnar cell layer, and a superficial columnar cell layer. In recent years, much progress has been made in regards to the exploration of adult epithelial homeostasis, cell turnover, and regeneration. Similar to other epithelial tissues such as the intestine (Tian et al. 2011; Haegebarth and Clevers 2009), skin (Blanpain et al. 2004; Tumbar et al. 2004), lung (Zuo et al. 2015; Kumar et al. 2011), bile duct (Parent et al. 2004; Cantz et al. 2008) and cornea (Meyer-Blazejewska et al. 2011; Rama et al. 2010), the basal cells in the cervix contribute to the epithelium tissue self-renewal under normal conditions, and this regeneration process can be accelerated by disruptions of the cervical barrier, such as excessive infection, inflammation, vascular insults, physical trauma or iatrogenic intervention.

Previously we have identified a SOX9 + P63 + KRT5+ airway basal cell population located at the bottom of rugaes in mouse and human airway which are capable to rapidly respond to lung injury and replenish damaged airway and alveolar tissue by switching from a homeostatic status to a regenerative status. These cells can be cloned and long-term propagated as clonal lineages derived from single cells in a feeder-based culture system in vitro, distinguishing from other non-clonogenic basal cells (Zuo et al. 2015; Ma et al. 2018).

Here we showed that a similar culture condition can be adapted to grow single-cell derived clones from cervical epithelium, and more importantly, the cloned cells demonstrate bi-lineage differentiation potential to give rise to either columnar or squamous cells. Therefore, we hypothesized that this clonogenic cell population of the cervix may be the stem/progenitor cells of the cervix, like their counterparts in other epithelial tissues and organs. To gain further insights into the features and behaviors of these putative stem/progenitor cells in vivo, the single-cell RNA-seq approach, which was frequently used to identify novel cell types or dissected cell heterogeneity within a ‘homogenous’ cell population (Wen and Tang 2016) in female reproductive system such as uterus (Wu et al. 2017), cervix (Chumduri et al. 2021) and vagina (Li et al. 2021), was also applied.

## Methods

### Mice and human tissues

All animal experiments were carried out in accordance with Chinese National Guidelines GB/T 35892–20,181, as well as under the guidance of the Institutional Animal Care and Use Committee of Tongji University. Mice were kept in a temperature-controlled SPF (Specific pathogen Free) environment with a regular light/dark cycle and provided with adequate rodent diet and water. All mouse experiments were performed on 8–10-week-old female mice and represent a minimum of *n* = 5 mice in all groups. All mice were randomly allocated to the experimental groups.

The human cervical specimens were obtained from women who underwent hysterectomies due to uterine leiomyomata in the Clinical Pathology Department of Shanghai First Maternity and Infant Hospital. A segment of tissue containing ectocervix, transformation zone, and endocervix, was taken after the removal of the uterus. The procedure was approved by the Shanghai First Maternity and Infant Hospital Ethics Committee. All the human tissues were collected following clinical SOP (Standard Operating Procedure) under the patient’s consent.

### Isolation and expansion of CVSC from the cervix

For CVSC isolation, the cervix was cut open and epithelium tissues were scraped off using the blade. Then pieces of specimens were washed with ice-cold wash buffer containing F12 (Life Technologies, USA), 5% FBS (Hyclone, USA), 20μg/mL Gentamicin (Sangon Biotech, China), and 1% Pen/Strep (Life Technologies, USA), followed by digestion with dissociation buffer including DMEM/F12 (Life Technologies, USA), 10% FBS (Hyclone, USA), 2 mg/mL protease XIV (Sigma-Aldrich, USA), 0.01% trypsin (Life Technologies, USA) and 10 ng/mL DNase I (Sigma-Aldrich, USA) in room temperature overnight with gentle agitation. Digested cell suspensions were washed 5 times with wash buffer and passed through a 70-μm Nylon mesh (Falcon, USA) to remove aggregates. Cell viability was assessed by the exclusion of trypan blue dye. Cell pellets were collected by centrifuge of 200×g and then seeded onto the non-lethally irradiated, growth-arrested 3 T3 cell feeder layer in modified SCM-6F8 medium (Wang et al. 2015). Typically, both mouse and human CVSCs are passaged every 5–8 days in a 1:8 ratio. For each mouse and human biological sample, the cell culture at P1 was assessed for cell immunofluorescence staining with p63 and KRT5 antibodies to confirm successful expansion of the putative CVSCs.

To obtain single cell-derived clones, cells were digested into single cells and seeded with extremely low density. A single colony derived from a single cell was picked up by a clone cylinder (Sigma, USA) and high vacuum grease (Corning, USA) after its neighboring cells were removed by a cell scraper to ensure the pedigree purity.

To determine the colony forming efficiency of cells at different passages, 1 hundred of alive CVSCs were randomly picked and single cells were paved on feeders in wells of 96-well plates. The single cell-derived clones in each well were counted after 7 days of culture. The colony forming efficiency (CFE) was calculated using the formula CFE = clone number / 100 × 100%. Movies recording the clone growth were captured using a Lonza CytoSMART™ System (Lonza, Switzerland).

### Histology and immunofluorescence

For cell immunofluorescence staining, cells were fixed by 4% paraformaldehyde, washed with PBS, and then incubated with 0.25% Triton X-100 for permeabilization. For tissue histology and immunofluorescence staining, tissue samples were fixed in 3.7% formaldehyde overnight at 4 °C. For cryosection, the fixed tissue was infiltrated with 30% sucrose before embedding, then embedded into the Tissue-Tek O.C.T compound (Sakura, USA), 5–8 μm sections were prepared using a cryotome (Leica microsystem, Germany).

For the paraffin section, the fixed tissue was dehydrated by ethanol gradient and processed in an automatic tissue processor (Leica microsystem, Germany), and then embedded into the paraffin blocks. All the samples were sectioned at 5–8 μm thickness using a microtome (Leica microsystem, Germany). Haematoxylin and eosin (H&E) staining was performed following standard protocol. Immunofluorescence staining was conducted to reveal the protein expression in a standard protocol using the antibodies on paraffin sections, cryosections, or glass smears.

Antibodies used for immunofluorescence include: KI67 (1:100, 550,609, BD bioscience), KRT5 (1:500, EP1601Y, Thermo Fisher Scientific), KRT17 (1:500, ab109725, Abcam), P63 (1:100, ab124762, Abcam), MUC1 (1:500, ab45167, Abcam), KRT10 (1:500, ab76318, Abcam), HUNU (1:200, ab191181, Abcam), GFP (1:500, ab6673,Abcam), KRT13 (1:500, ab92551, Abcam), CDH1 (1:500, 3195S, Cell Signaling), PAEP (1:500, ab17247, Abcam), IVL (1:500, ab53112, Abcam), KRT8 (1:500, ab217173, Abcam), KRT1 (1:500, ab93652, Abcam), KRT14 (1:500, ab7800, Abcam), and human specific LAMIN A + C (1:500, ab108595, Abcam). Alexa Fluor-conjugated Donkey 488/594 (1:500, Life Technologies, USA) were used as secondary antibodies. In control experiments, the primary antibodies were replaced by IgG. All the antibodies were tested for efficacy and specificity before use. Quantitative analysis of the images for all outcome measures was conducted using ImageJ software (version 1.48).

### Quantitative reverse transcription PCR

Total RNA from tissues or cells were isolated using the RNeasy mini kit with DNase digestion according to the manufacturer’s instructions (Qiagen, Germany). RNA quality was determined by SimpliNano (GE Healthcare, USA). One microgram total RNA was reverse-transcribed into cDNA with PrimeScript™1st Strand cDNA synthesis Kit (TaKaRa, Japan). The real-time PCR assays were performed on an ABI 7500 real-time PCR system (Applied Biosystems) according to the instructions of SYBR® PremixEx Taq™II (Tli RNaseH Plus, Takara). qPCR reactions were set as following: 95 °C for 2 min, then 40 cycles of 95 °C for 10 s, and 60 °C for 40 s. Melt curve stage was added after PCR amplification stage. The threshold crossing value (Ct) of each transcript was normalized to reference gene GAPDH. The relative expression level of each genes was calculated using the 2^−ΔΔCt^ method. Sequence of primer pairs for qPCR was listed in Supplementary Table 1.

### Cell karyotyping

To arrest human CVSC in mitosis metaphase, cells at 75% confluence were treated with 1 μg/mL colchicines for 7 h and digested into single cells by 0.25% trypsin (Gibco, USA). Then the cells were incubated with 0.4% KCl at 37 °C for 40 min and fixed by 10 mL fixation solution including methanol and glacial acetic acid (3:1) at room temperature for 30 min. The prepared cell suspension was dropped and spread on slides. Samples on slides were treated with 0.0005% trypsin for 5 min and stained with 15% Giemsa (Sigma-Aldrich, USA). Banding patterns on chromosome spreads were checked for more than 15 mitotic phases.

### Mouse cervix injury models

To establish a chemical damage model of the mouse cervical epithelium, 8–10-week-old C57/BL6 mice were anesthetized by intraperitoneal injection of 4% chloral hydrate (0.5 g/kg body weight, Sigma-Aldrich, USA). Fifty microlitre of two point five percent TCA (Trichloroacetic acid, 76–03-9, Aladdin, China) was injected into the cervix using a syringe (Darwish and Zahran 2013; Malviya et al. 1987; Menéndez Velázquez et al. 1993). Ten minutes later, the cervix was washed by sterile PBS for 3 times. Mice were euthanized after injury at indicated time points and cervix samples were harvested for further analysis.

### Lineage tracing experiments

Krt5 Cre^ERT2^-Gt (ROSA)26Sor^tm4(ACTB-tdTomato-EGFP)^ mice were used to perform lineage tracing. For induction of Cre-ERT2 protein, 8–10-week-old mice were injected with tamoxifen dissolved in corn oil via an intra-peritoneal route. For each mouse, 0.1 mg tamoxifen was used to label single cells (Kusaba et al. 2014; Kang et al. 2016). Seven days after the last injection of tamoxifen, mice were TCA injured. Mice were euthanized after injury at indicated time points and cervix samples were harvested for further analysis.

### 3-dimensional culture of CVSC spheroids

3D culture of cells was performed on the Matrigel Matrix (Corning, USA) as previously described (Zuo et al. 2015). For CVSC spheroid formation, 24 h after cell seeding, the medium was changed to cervical 3D differentiation medium including Advanced DMEM/F12 (Life Technologies, USA), 1% pen/strep (Life Technologies, USA), 1X HEPES (Life Technologies, USA), 1X Glutamine (Life Technologies, USA), 1 mM N-acetylcysteine (Sigma-Aldrich, USA), 1% N2 (Life Technologies, USA), 1% B27 (Life Technologies, USA), 10 μM ROCK inhibitor Y-27632 (M.C.E., China), 50 ng/ml EGF (Preprotech, USA), 100 ng/ml FGF 10 (Preprotech, USA) and 10 mM nicotinamide (M.C.E., China), as described in previous reports (Maru et al. 2020; Sato et al. 2011; Chumduri et al. 2018). The spheroids were then cultured in the differentiation medium until harvest. The harvested samples were subjected to immunofluorescence and bulk RNA-seq analysis. For immunofluorescence, 5 μm cryosections were prepared and stained with squamous and columnar epithelium markers followed by counterstaining with 4′,6-Diamidino-2-Phenylindole, Dihydrochloride (DAPI).

### Mouse renal capsule transplantation

Eight to ten week-old immune-deficient NOD-SCID mice were anesthetized by intraperitoneal injection of 4% chloral hydrate (0.5 g/kg body weight, Sigma-Aldrich, USA). The left lateral peritoneum was cut to expose the left kidney. CVSCs were injected into the renal capsule. For each mouse, 1 × 10^6^ human CVSCs were used. Mice were euthanized 14 days after the transplantation and the kidney samples were harvested for further analysis.

### Bulk RNA-sequencing analysis and bioinformatics

Mouse cervical tissues, CVSCs, and spheroids were analyzed by bulk RNA-sequencing. Duplicate experiments were taken from 2 biological samples. RNA was extracted using RNeasy® Mini Kit (REF# 74104) and a total amount of 3 μg RNA per sample was used as input material for the RNA sample preparations. Sequencing libraries were generated using NEBNext® Ultra™ RNA Library Prep Kit for Illumina® (NEB, USA) following the manufacturer’s recommendations and index codes were added to attribute sequences to each sample. Raw data (raw reads) of FASTQ format were firstly processed through in-house PERL scripts. In this step, clean data (clean reads) were obtained by removing reads containing adapter, reads containing ploy-N and low-quality reads from raw data. All the downstream analyses were based on the clean data with high quality. FASTQ files were aligned against the mouse reference (mm10) genome. Index of the reference genome was built using STAR (v2.5.1b) and paired-end clean reads were aligned to the reference genome. HTSeq v0.6.0 was used to count the reads numbers mapped to each gene, and then FPKM (FragmentsPer Kilobase per Million) of each gene was calculated based on the length of the gene and read counts mapped to this gene. Differential expression analysis of two conditions/groups (two biological replicates per condition) was performed using the DESeq2 R package (1.10.1). The resulting *P*-values were adjusted using the Benjamini and Hochberg’s approach for controlling the false discovery rate. Genes with an adjusted P-value < 0.05 found by DESeq2 were assigned as differentially expressed. Corrected P-value of 0.05 and an absolute fold change of 2 were set as the threshold for significant differential expression. Relative expressions of essential markers were displayed by GraphPad Prism (version 8.0). R package pheatmap was used to create all heatmap graphs in this analysis.

### Gene set enrichment analysis

Gene Set Enrichment Analysis (GSEA) of differentially expressed genes in bulk RNA-Seq data were implemented by the ClusterProfiler R package (Yu et al. 2012). For Wnt and EGFR gene analysis, the hallmark gene sets in MsigDB (Liberzon et al. 2015) (msigdb.v7.4.symbols.gmt and c3.all.v7.4.symbols.gmt) were used for annotation. The enrichplot R package was used to visualize the distribution of the gene set and the enrichment score.

### Single-cell RNA sequencing analysis

Single cells were captured and barcoded in 10x Chromium Controller (10x Genomics). Subsequently, RNA from the barcoded cells was reverse-transcribed and sequencing libraries were prepared using Chromium Single Cell 3’v2 Reagent Kit (10x Genomics) according to manufacturer’s instructions. Sequencing libraries were loaded on an Illumina NovaSeq with 2 × 150 paired-end kits at Novogene, China. Raw sequencing reads were processed using the Cell Ranger v.3.0.0 pipeline from 10X Genomics. In brief, reads were demultiplexed, aligned to the mouse mm 10 genome and UMI counts were quantified per gene per cell to generate a gene-barcode matrix. Data were aggregated and normalized to the same sequencing depth, resulting in a combined gene-barcode matrix of all samples. Seurat v.3 was used for quality control, dimensionality reduction, and cell clustering. The low-quality cells with less than 200 or more than 6000 detected genes were removed, or if their mitochondrial gene content was > 10%. Genes were filtered out that were detected in less than 3 cells. This filtering step resulted in 15,361 genes X 4752 cells. The filtered gene-barcode matrix was first normalized using ‘LogNormalize’ methods in Seurat v.3 with default parameters. The top 2000 variable genes were then identified using the ‘vst’ method in Seurat FindVariableFeatures function. Variables ‘nCount_RNA’ and ‘percent.mito’ were regressed out in the scaling step and PCA was performed using the top 2000 variable genes. Then UMAP (uniform manifold approximation and projection) was performed on the top 50 principal components for visualizing the cells. Meanwhile, graph-based clustering was performed on the PCA-reduced data for clustering analysis with Seurat v.3. The resolution was set to 0.3 to obtain a finer result. The immune cell cluster was removed and the other cells were re-clustered using the same parameter mentioned above in the clustering step and parameter resolution was set to 0.3. MAST (Finak et al. 2015) in Seurat v.3 (FindAllMarkers function) was used to perform differential gene expression analysis. For each cluster of epithelial cells, DEGs were generated relative to all of the other cells. A gene was considered significant with adjusted *P* < 0.05 (*P* values were adjusted by false discovery rate in MAST). Violin plots were performed by the function VlnPlot in Seurat with the default parameters. Expression heatmap for marker genes was performed by the function DoHeatmap in Seurat with the default parameters.

### Cell cycle analysis

A cell cycle score was assigned on each cell according to its gene expression of G2/M and S phase markers based on the scRNA-seq data. Based on this scoring system, each cell was classified in either G2/M, S, or G1 phase using the CellCycleScoring function in Seurat. The cells at different cell cycle classifications were visualized in the UMAP map.

### Cell type specific expression of disease-associated genes

To calculate the average expression level for each cluster in scRNA-seq data, the function AverageExpression in Seurat was used with the default parameters. To plot the disease-associated genes, R package pheatmap was used to create all heatmap graphs in this analysis.

### Cell-cell communication analysis

Cell-cell communication was predicted based on the scRNA-seq data by using CellPhoneDB (Vento-Tormo et al. 2018) version 2.1.2 (https://github.com/Teichlab/cellphonedb). Only receptors and ligands expressed in at least 10% of cells of a given cell type were further analyzed, whereas the interaction was considered nonexistent if either the ligand or the receptor was undetectable. The average expression of each ligand-receptor pair was compared between different cell types, and only those with *p* < 0.05 were used for subsequent prediction of cell-cell communication in different cell types. Network visualization was done using Cytoscape (version 3.2.0). The network layout was set to the force-directed layout.

### Single cell trajectory analysis

To infer the cluster and lineage relationships between the different epithelial cell types identified in scRNA-seq data, Monocle3 (Qiu et al. 2017; Cao et al. 2019) was used (https://github.com/cole-trapnell-lab/monocle3). UMAP embeddings and cell clusters generated from Seurat were used as input, and trajectory graph learning and pseudo-time measurement through reversed graph embedding was performed with Monocle3. To construct single cell pseudo-time trajectory and to identify genes that change as the cells undergo transition, Monocle2 (Trapnell et al. 2014) (version 2.4.0) algorithm was also applied to our data. Genes for ordering cells were selected if they were expressed in ≥10 cells, their mean expression value was ≥0.1 and dispersion empirical value was ≥2. Cells were ordered along the trajectory and their trajectory was visualized on the reduced dimensional space. Significantly changed genes along the pseudo-time were identified using differential GeneTest function of Monocle2 with q-value < 0.01.

### Gene Ontology (GO) analysis

GO biological process and pathway enrichment analyses of differentially expressed genes in scRNA-seq data were performed using Metascape (Zhou et al. 2019) (version 3.5) (http://metascape.org), and the results were visualized with the ggplot2 R package. GO terms with a *P* value less than 0.01 were considered significantly enriched by differentially expressed genes.

### Comparison of the single cell with bulk RNA sequence data

Our mouse cervix single cell data was correlated with bulk RNA sequencing data of single cell colonies (A and B). DEGs (identified as described above) were used for correlation analysis. Z-scores were calculated for normalized expression values of each gene in single cell data and for FPKM values of each gene in bulk RNA sequencing data. Subsequently, Pearson-correlation coefficients were calculated between every pair of single cell data and replicates of bulk RNA sequencing data.

### Statistical analysis

Block randomization was used to randomize mice and samples into groups of similar sample sizes. No data was intentionally excluded. Postoperative deaths of animals were excluded from the study. For the immunofluorescence histochemistry analysis, all experiments were assessed by at least two blinded participating investigators. Results are presented as means ± SD. GraphPad Prism (version 8) or R programming were used for data management, statistical analysis, and graph generation. The exact number (n) of sample size for each experiment was stated in the corresponding figure legend. Differences with *P* ≤ 0.05 were considered statistically significant.

## Results

### Identification of P63+/KRT5+ CVSCs cloned from mouse and human cervix

**Fig. 1:**
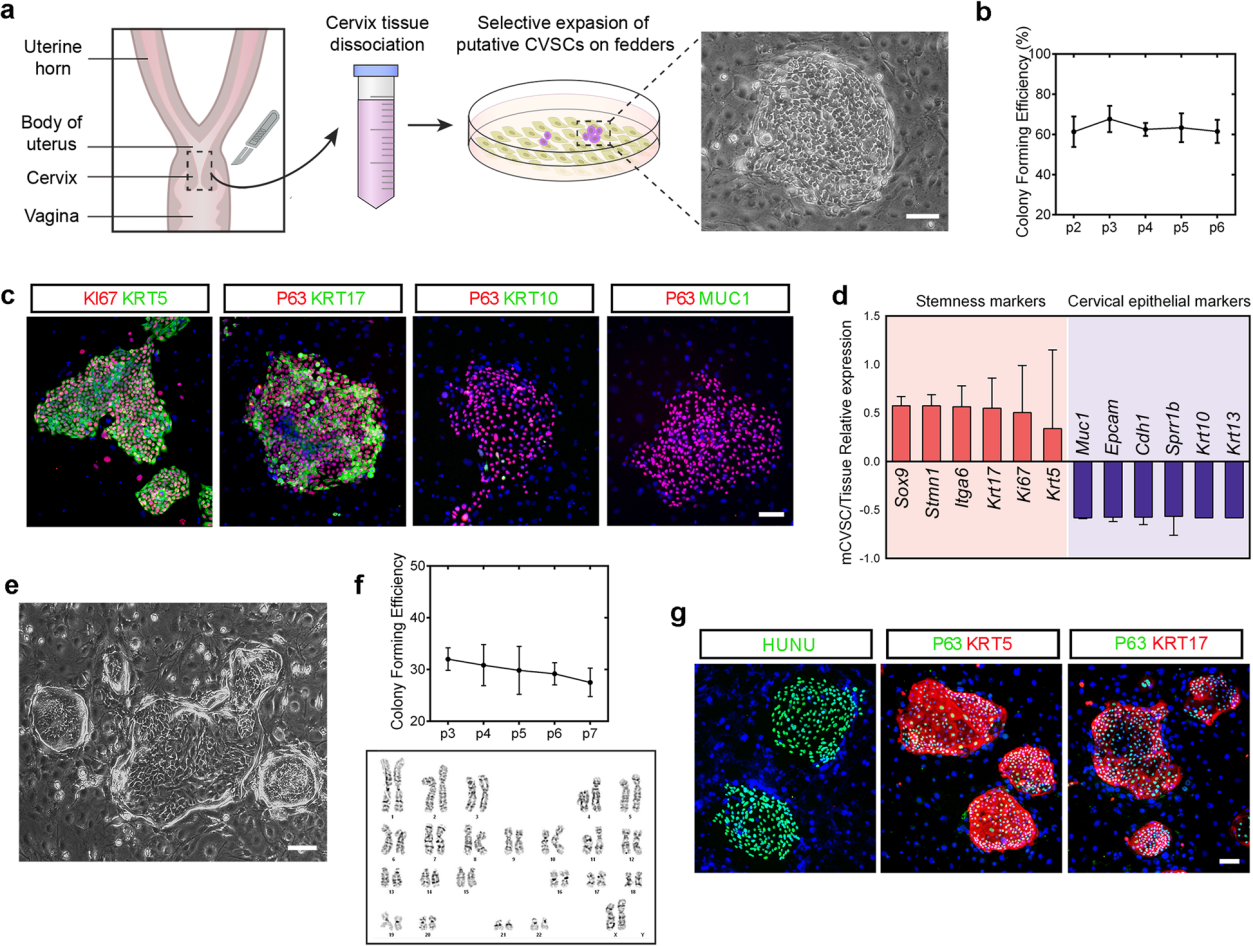
Cloning and identification of murine and human CVSCs. **a** Schematic shows the selective culture of putative CVSCs from the mouse cervix and representative phase-contrast microscopic image presenting primary cell colony. Scale bar, 100 μm. **b** Colony forming efficacy of CVSCs of different culture passages. Data representative of *n* = 3 biological replicates. p, passage. **c** Putative mouse CVSC colonies stained with proliferative marker KI67, cervical epithelial stem cell markers KRT5, KRT17, and P63, cervical squamous cell marker KRT10, and cervical columnar cell marker MUC1. Data representative of *n* = 10 biological replicates. Scale bar, 100 μm. **d** Relative expression levels of genes frequently upregulated in epithelial stem cells and genes considered as differentiated cervical epithelial markers. **e** Representative phase-contrast microscopic image of putative human CVSC colonies. Scale bar, 100 μm. **f** Colony forming efficacy and representative karyotype of human CVSCs. Cells for karyotyping were harvested at passage 15. Data representative of *n* = 3 biological replicates. p, passage. **g** Putative human CVSC colonies stained with human-specific marker human nuclear antigen (HUNU), and adult epithelial stem cell markers KRT5, KRT17, and P63. Data representative of *n* = 3 biological replicates. Scale bar, 100 μm

We introduced a feeder cell-based cloning system to achieve culture of putative stem cells from the cervix. Previously we successfully used this cell culturing protocol to isolate and expand P63+/KRT5+ distal airway stem cells from the lung (Zuo et al. 2015). In this culture system, only those proliferating cells with clonogenic potential can grow. The emerged mouse cell aggregates exhibiting typical epithelial stem/progenitor clone morphology on the feeder layer within 3 ~ 7 days after seeding (Fig. 1a). The obtained clonogenic cells could be passaged with stable clonogenicity (Fig. 1b), demonstrating the robustness of the culture system for stable cell self-renewal in vitro. Immunostaining further confirmed that all the emerged clonogenic cells are positive for typical epithelial stem/progenitor cell markers P63, KRT5, KRT17, and proliferative cell marker KI67, and negative for the mature squamous or columnar cell markers (KRT10 and MUC1) which are expressed in the cervical epithelium (Fig. 1c and S1a). To characterize this clonogenic epithelial cell population derived from the mouse cervix, bulk RNA-sequencing (RNA-seq) analysis was performed between the cultured cell clones and the corresponding cervix tissues. Genes expressed in epithelial stem/progenitor cells (*Sox9, Stmn1, Itga6, Krt17, Ki67,* and *Krt5*) were highly expressed compared to the cervix tissue, while expressions of columnar epithelial markers (*Muc1, Epcam, and Cdh1*) and squamous epithelial markers (*Sprr1b, Krt10, and Krt13*) were maintained at low levels in the clonogenic cells (Fig. 1d). Genes expression of mouse CVSC (mCVSC) and the corresponding cervix tissues was further confirmed by qPCR analysis (Fig. S1c and S1d).

Our successful isolation of the clonogenic cervical cells from the mouse cervix encouraged us to further test the culture system to grow such cells from human. The human cervix specimens were obtained from women who underwent hysterectomies and the digested single-cell suspensions were seeded for cell culture. All of the samples successfully yielded cell colonies within 10 days (Fig. 1e). The clonogenicity of the cultured human cells was stably maintained for at least 20 passages. Additionally, cell karyotyping at Passage 15 demonstrated that cells retain a normal number of chromosomes over a long-term culture period (Fig. 1f). The human cell colonies grown on mouse feeder cells are positive for human-specific nuclei antigen HUNU, as well as KI67, P63, KRT5, and KRT17 (Fig. 1g and S1b).

**Fig. 2:**
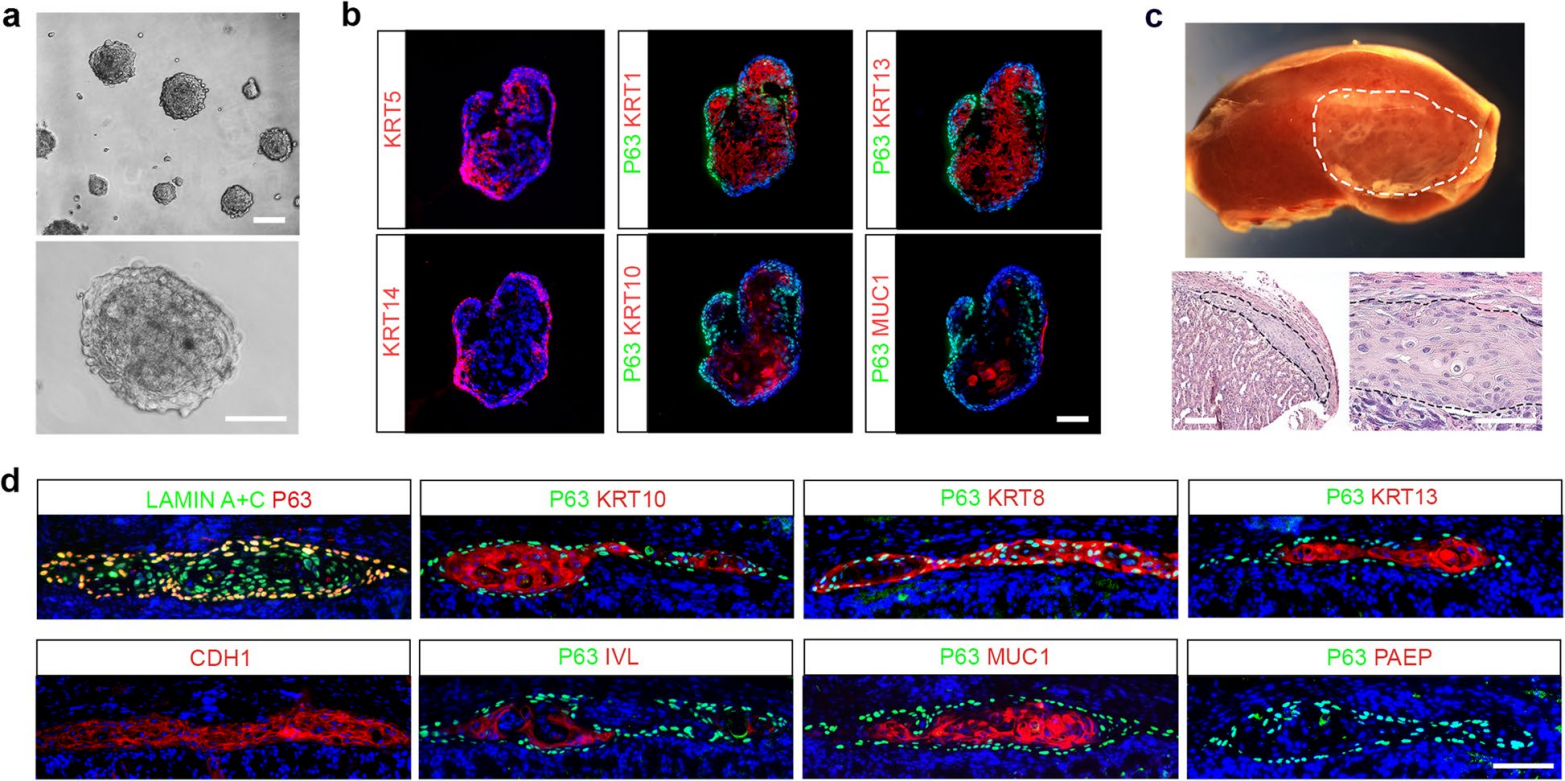
In vitro and in vivo differentiation of CVSCs. **a** 3D culture of mouse CVSCs on the Matrigel Matrix. Scale bars: 100 mm. **b** Immunofluorescence on sections of the spheroids with indicated cervical markers on Day 14. Data representative of *n* = 3 biological replicates. Scale bar, 100 μm. **c** Upper: Structures generated from subcapsular transplantation of human CVSCs into the kidneys of NOD-SCID mice. Lower: H&E staining of the transplanted area 14 days post-injection. Dashed outline indicates transplanted human CVSCs. Scale bar, 100 μm. **d** Immunofluorescence on sections of transplanted human CVSCs with indicated cervical epithelial markers. Data representative of *n* = 3 biological replicates. Scale bar, 100 μm

To evaluate the differentiation potential of clonogenic KRT5 + P63+ cells in vitro, 3D culture on Matrigel was used to support differentiation of mouse cervical cells. The clonogenic KRT5 + P63+ cells spontaneously aggregated and formed cell spheres 24 h after seeding (Fig. 2a). After 3D differentiation for 14 days, the formed cell spheroids demonstrated spatial heterogeneity indicated by marker expression of different cell types. Cells expressing KRT5, KRT14, and P63 were located at the basal layer of the spheroids, while differentiated cells expressing KRT1, KRT13, KRT10, and MUC1 were present at the surface of the luminal area (Fig. 2b), suggesting both squamous and glandular differentiation of the clonogenic KRT5 + P63+ cells in vitro.

To further determine the differentiation capacity of human cervix-derived cells in vivo, we xenografted human clonogenic KRT5 + P63+ CVSCs under the mouse renal capsule. After 14 days of differentiation in vivo, H&E staining showed the stratified epithelial structure of the transplanted cells (Fig. 2c). Immunostaining in Fig. 2d confirmed the expression of human-specific marker (human LAMIN A + C), epithelial cell marker (CDH1), squamous epithelial markers (KRT10, KRT13, and IVL), and glandular columnar epithelial markers (KRT8 and MUC1), providing additional evidence that the expanded human CVSCs maintain their differentiation potential during culture. Altogether the above data have revealed that the clonogenic KRT5 + P63+ CVSCs derived from human and mouse cervix are bipotent, which are capable to differentiate into either columnar or squamous cells in vitro or in vivo.

### KRT5+ CVSCs reconstitute cervical epithelium during homeostasis and injury repair

In the cervix, the CVSCs exist underneath the cervical epithelium and distribute from the ectocervix to the distal part of the endocervix. To understand whether the CVSCs in situ could replenish the lost cells in the cervical epithelium, we used a genetic lineage tracing mouse model to study this process. In these Krt5 Cre^ERT2^-Gt(ROSA)26Sor^tm4(ACTB-tdTomato-EGFP)^ transgenic mice, we used an extremely low dose of tamoxifen (0.1 mg per mouse) to label only a few single KRT5+ cells and traced their fate 10 days post labeling. The result showed that in the ectocervix, the genetically labeled cells gave rise to multiple layers of KRT13+ squamous cells; while in the endocervix, the labeled cells gave rise to MUC1+ columnar cells (Fig. S2a). These data indicated that in homeostatic status, CVSCs could contribute to the maintenance of daily turnover of cervical epithelium.

**Fig. 3:**
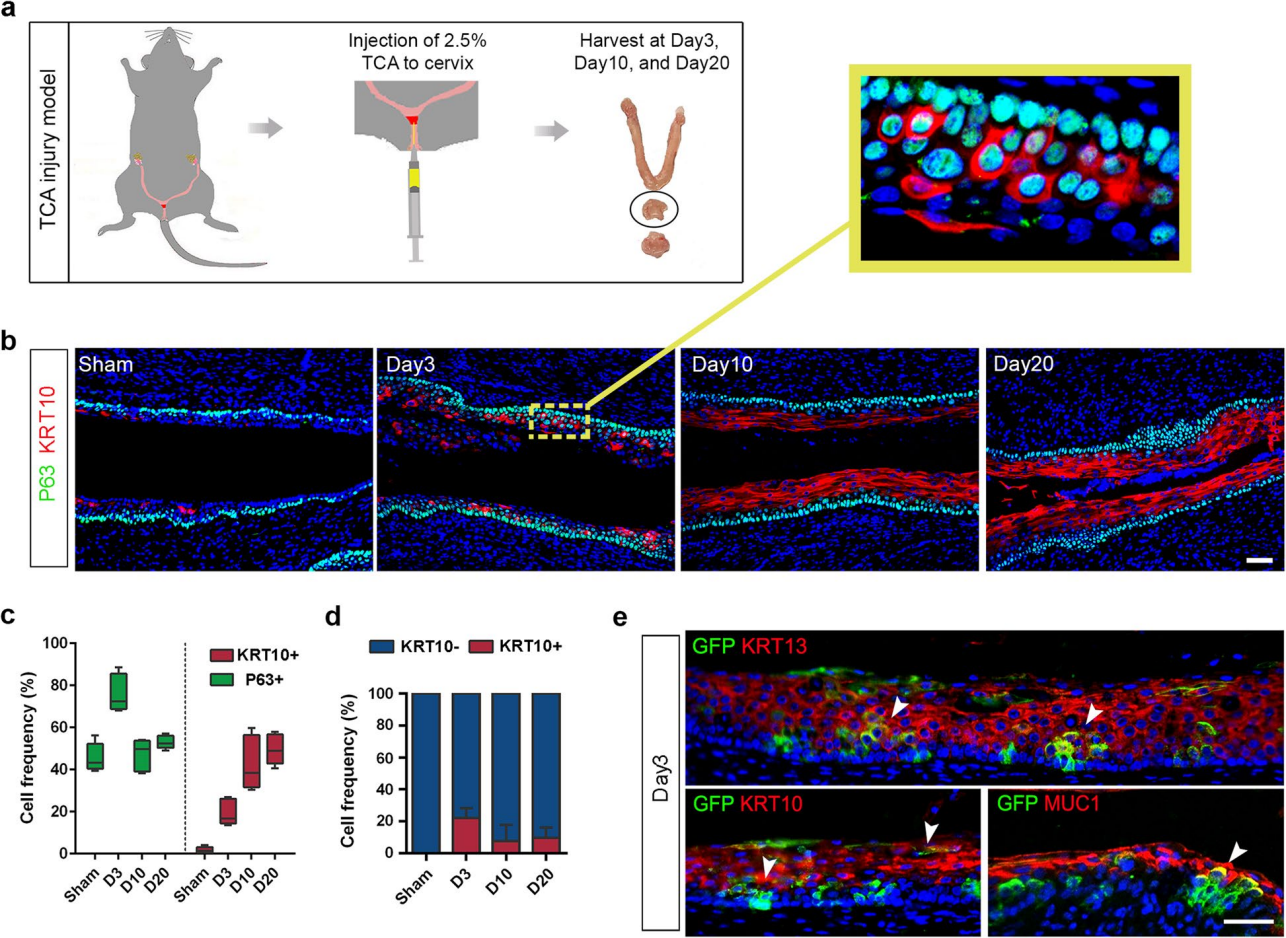
Cervix epithelium regeneration in the TCA injury model. **a** Experimental scheme. 6–8-week-old female mice were treated with 2.5% TCA via direct injections into the cervix, and the cervix samples were collected at three time points post-injection (Day3, Day10, and Day20) for histological analysis. Data representative of *n* = 5 biological replicates. **b** Expression of P63 and KRT10 along the endo-cervical epithelium at indicated days after TCA injury. Enlarged inset outlined by yellow: P63 + KRT10+ cells showing initiation of squamous metaplastic formation after injury. Sham cervix served as a negative control. Data representative of *n* = 5 biological replicates per time point. Scale bar, main panel, 100 μm; enlarged inset,10 μm. **c** P63+ and KRT10+ cell frequency in the injured endo-cervix epithelium. Data representative of *n* = 5 biological replicates per time point. **d** KRT10- and KRT10+ cell fraction in P63+ cells at designated post-injury time points was plotted as mean ± SD. Data representative of *n* = 5 biological replicates per time point. **e** Lineage tracing experiments in Krt5 Cre^ERT2^-Gt(ROSA)26Sor^tm4(ACTB-tdTomato-EGFP)^ mice treated with low-dose tamoxifen prior to TCA injury reveals that squamous metaplasia arising after the injury is positive for Krt5-GFP lineage marker on Day3. Data representative of *n* = 5 biological replicates. Scale bar, 100 μm

Next, we analyzed whether such CVSCs could contribute to the damage repair process of the cervix. We established a chemical injury mouse model induced by direct injection of 2.5% trichloroacetic acid (TCA) to the cervix, which mimics the chemical-based clinical surgery commonly used in gynecology (Fig. 3a). TCA application severely damaged the upper layers of epithelium for both ectocervix and endocervix. For the endocervix, massive squamous metaplasia occurred after the injury that a large part of the endocervical columnar epithelium was transformed to the stratified epithelium. As a result, 3 days post-injury, we observed an elevated number of P63+ CVSCs and KRT10+ squamous cells in the transformation zone (TZ) compared to the sham group by immunofluorescence. Many cells in differentiating status co-expressing both P63 and KRT10 markers were observed at this time point. As expected, the proportion of KRT10+ cells gradually increase 10 days and 20 days post-injury, while the P63+/KRT10+ co-expression proportion peaks at 3 days post-injury, indicating the formation of stratified squamous epithelium by P63+ CVSCs (Fig. 3b-d).

To determine whether CVSCs actively contribute to cervical epithelial repair, we performed in vivo genetic fate mapping of KRT5+ cells in the TCA injury model. After low-dose tamoxifen (0.1 mg/mice) labeling of GFP+ single cells, mice were then subjected to sham or TCA cervix injury. The results showed that the genetically labeled CVSCs could give rise to a large amount of mature KRT13+ /KRT10+ squamous cells in transformation zone after damage, and there were also a few MUC1+ glandular cells that were regenerated (Fig. 3e on Day3 and S2b on Day 10).

### Single-cell atlas of the cervix epithelium

**Fig. 4:**
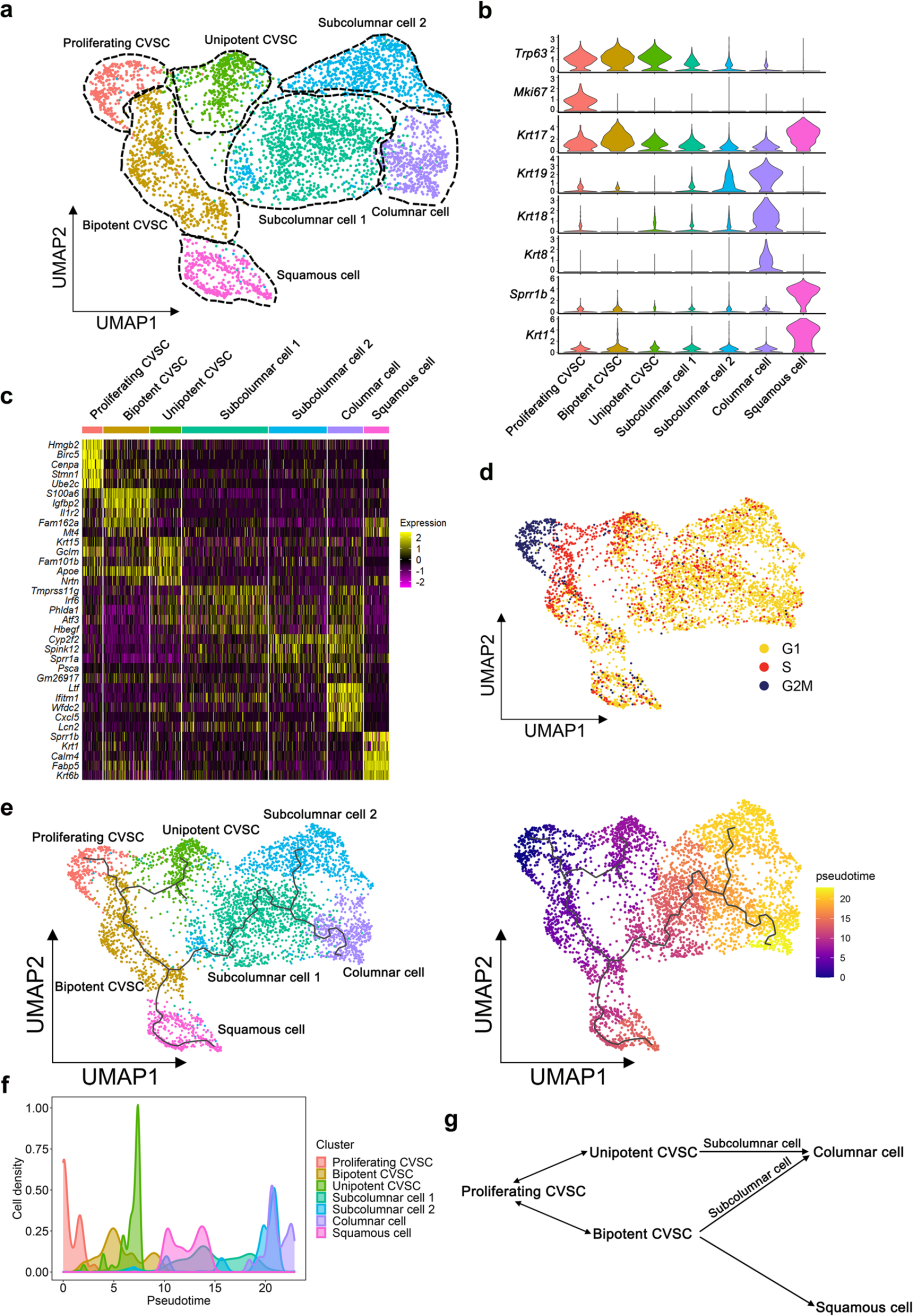
Identification of distinct cell types of the mouse cervix epithelium by single-cell RNA sequencing. **a** 7 distinct cell clusters from 10x Genomics scRNA-seq analysis visualized by UMAP. Each dot represents a single cell. Colors and numbers indicate clusters, and cell-type names are indicated with their corresponding clusters. **b** Violin plots showing the expression levels of representative marker genes across the 7 clusters. The y axis shows the log-scale normalized read count. **c** Heatmap of the top-ranked genes highly expressed in each cluster. Color scheme is based on z-score distribution from − 2 (purple) to 2 (yellow). **d** UMAP plot showing the cell cycle status of each cell. **e** Pseudo-time trajectory projected onto a UMAP of cervical epithelial cells. Left, distinct cell clusters are coded by different colors; Right, pseudo-time values are color-coded. **f** Cell density of cervical epithelial cell clusters inferred cellular trajectory reflecting cervical epithelial cell differentiation. Cells are ordered by pseudo-time as computed by reversed graph embedding approach of Monocle3. **g** Schematic shows the putative differentiation paths from CVSCs to columnar cells or squamous cells predicted by the pseudo-time analysis

The above studies based on cultured cells and in vivo lineage tracing are all based on a population of CVSCs instead of single cells. It remains unknown that what type of differentiation potential a single stem cell could have. To illustrate the cell composition of the mouse cervix, 10X genomics single-cell RNA sequencing was performed. We isolated and sequenced a total of 5607 cells, and eventually analyzed 4669 cells after stringent quality control and removing the immune cell cluster (Fig. S3). We performed unsupervised clustering analysis on the other cells, and finally visualized transcriptionally distinct cell clusters using uniform manifold approximation and projection (UMAP) (Fig. 4a). Unsupervised clustering identified 7 transcriptionally distinct populations present in the mouse cervix. To define the identity of each cell cluster, we generated cluster-specific marker genes by performing differential gene expression analysis (Fig. 4b and c). Cell clusters were identified by known cell type-specific markers, including *Krt8* and *Krt18* for columnar epithelial cells, *Sprr1b* and *Krt1* for squamous epithelial cells, and *Trp63* for three CVSC clusters. The CVSC clusters included a *Krt17*-high CVSC subpopulation, a *Krt17*-low subpopulation and a *Mki67*+ subpopulation (proliferating CVSCs) (Fig. 4b). To understand the cervical epithelium cell cycle, we performed the cell cycle analysis of the single cells (Fig. 4d). The result showed that almost all proliferating CVSCs (99.4%) and approximately half of *Krt17*-high CVSCs and *Krt17*-low CVSCs (44.5% and 50.4%) are in the G2/M or S phase of the cell cycle, suggesting that three types of CVSC are the most active cycling cell population in mouse cervix. Altogether, our single-cell transcriptomic atlas provides a molecular definition of two previously defined cervix cell types, as well as three types of CVSC.

### Two types of CVSC represent different biological lineages in the cervix

Since our scRNA-seq data has indicated the existence of CVSC subpopulations, to understand their correlations and the differentiation continuum in the cervix, we further computationally inferred differentiation trajectory of CVSCs from the scRNA-seq atlas by pseudo-time mapping using reversed graph embedding method. We found that the *Krt17*-low CVSCs could differentiate into only columnar cells; in contrast, the *Krt17*-high CVSCs could differentiate into both squamous cells and columnar cells (Fig. 4e-g). We also found similar results with another analytical tool, Monocle2 (Fig. S4a and S4b). Therefore, the previously defined *Krt17*-low CVSCs could represent a “unipotent” stem/progenitor cell population while the *Krt17*-high CVSCs could represent a “bipotent” stem/progenitor cell population. Immunostaining also confirmed the existence of two subpopulations of *Trp63*+ CVSCs (Fig. S4c). The current discovery about CVSC subtypes is highly consistent with a previous report by the Hopman group, that there is an enrichment of *Krt17*-positive subpopulation in endocervical cells having a progenitor cell function for both squamous and columnar epithelium, and a subpopulation of *Krt17*-negative cells having only a progenitor cell function for columnar cells (Martens et al. 2009).

We next explored the biological implications of genes whose expression levels were significantly up-regulated in bipotent CVSCs and unipotent CVSCs using Gene Ontology (GO) and pathway analyses (http://metascape.org/). The results showed that the up-regulated genes in bipotent CVSCs were enriched in tissue regeneration, cell junction organization, and formation of the cornified envelope. In contrast, up-regulated genes in unipotent CVSCs were enriched in the TGF-beta signaling pathway, Wnt signaling pathway, and gland development (Fig. S4d). Taken together, these data revealed that the bipotent CVSCs and unipotent CVSCs could have distinct differentiation potentials.

### Cervix epithelial lineage dynamics reconstructed by single-cell clone 3D differentiation

**Fig. 5:**
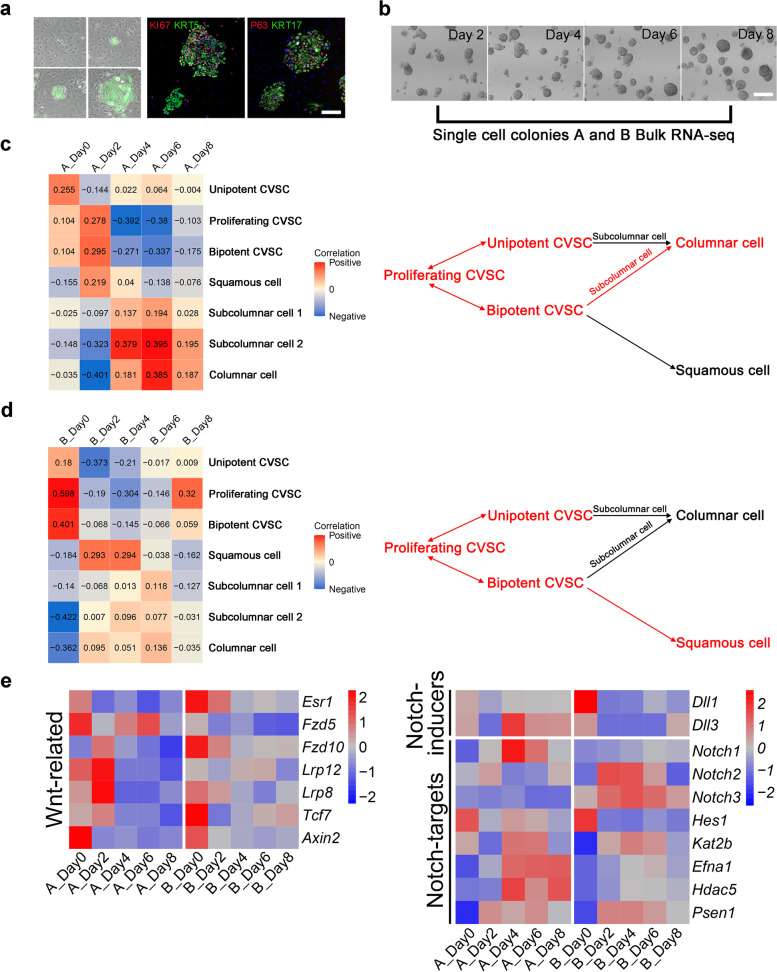
Cervical epithelium differentiation reconstruction by single-cell clone culture. **a** Representative phase-contrast microscopic image of GFP+ mouse CVSC single-cell colonies. Scale bar, 100 μm. Mouse CVSC single-cell colonies stained with proliferative marker KI67, and adult epithelial stem cell markers KRT5, KRT17, and P63. Data representative of *n* = 3 biological replicates. Scale bar, 100 μm. **b** 3D differentiated single-cell colonies A and B at 2, 4, 6 and 8 days since 3D culture obtained for bulk RNA sequencing. Scale bars, 100 mm. **c** The heatmap showed the Pearson correlation coefficient between single-cell colony A and bulk RNA sequence data. Each row represents one cluster from single-cell RNA sequence data and each column represents one single cell clone-specific bulk RNA-seq sample (left). Schematic shows alignment of single-cell colony A bulk RNA-seq data from 5 in vitro time points projected onto the pseudo-time differentiation map. The alignment of bulk RNA-seq data is shown in the red line (right). **d** The heatmap showed the Pearson correlation coefficient between single-cell colony B and bulk RNA sequence data. Each row represents one cluster from single-cell RNA sequence data and each column represents one single cell clone-specific bulk RNA-seq sample (left). Schematic shows alignment of single-cell colony B bulk RNA-seq data from 5 in vitro time points projected onto the pseudo-time differentiation map. The alignment of bulk RNA-seq data is shown in the red line (right). **e** Expression analysis of differentially regulated genes in mouse CVSC single-cell colonies at different days after aggregation and culture

To test whether the single-cell pseudo-time mapping indeed captures the real paths corresponding to cervix epithelial differentiation trajectory, we compared the computationally reconstructed pseudo-time map to the “real-time” differentiation according to a mouse in vitro single-cell differentiation assay. Two single-cell derived mouse CVSC clones (named A and B, respectively) were selected for 3D spheroid differentiation experiments (Fig. 5a). We performed bulk RNA sequencing on the 3D differentiated single-cell clone A and B at 0, 2, 4, 6, and 8 days since differentiation (Fig. 5b). Then the identity of cells at a given time point was defined by correlation calculation with single-cell transcriptomic data. For clone A, the results showed that it potentially represented a columnar lineage differentiation dynamics from Day 0 to Day 8 (Fig. 5c), whereas clone B followed a squamous differentiation (Fig. 5d). These data confirmed that our current culture system could support the large-scale expansion of bipotent CVSCs with both columnar and squamous differentiation potentials, and showed that the real-time observation indeed recapitulated the predicted cell differentiation roadmap made by pseudo-time analysis.

Next, we sought to identify the signaling pathways that control the bi-lineage differentiation of CVSCs. Based on the pseudo-time analysis, we found that Notch-related genes *Notch2*, *Notch3* and, *Jag1* as well as columnar cell marker *Krt8* were all upregulated in cells following the columnar epithelium path, compared to those following Krt10+ squamous epithelium paths (Fig. S5a). Furthermore, RNA-seq analysis of single-cell colonies A and B at different days after aggregation and culture showed that multiple Wnt-related genes were upregulated in undifferentiated CVSCs. A survey of genes upregulated in single-cell clone B versus differentiated cells revealed high expression of the Notch ligands delta-like ligand 1 (*Dll1*). By contrast, differentiated cells expressed higher levels of *Notch2* and *Notch3* receptors and their targets, including presenilin 1 (*Psen1*), a core component of γ-secretase (Fig. 5e). Together, these observations suggest the molecular mechanisms that the Wnt-related pathway could maintain the self-renewal of CVSCs (Clevers et al. 2014; Farin et al. 2012) and the Notch-related pathway induced cell maturation, especially epithelium differentiation (VanDussen et al. 2012; Noah and Shroyer 2013).

Next, gene set enrichment analysis (GSEA) also demonstrated that the expression of the hallmark Wnt gene list in Molecular Signatures Database (MSigDB) was significantly enriched in undifferentiated single-cell colony A (*P* = 0.083) and the expression of the hallmark EGFR gene list was significantly enriched in undifferentiated single-cell colony B (*P* = 0.039) (Fig. S5b). Further, GSEA revealed that target genes regulated by transcription factors downstream of EGFR signaling, were high enriched in undifferentiated single-cell colony B (Fig. S5c). These pathways function together to regulate proliferation and differentiation (Tsuda et al. 2002; Avraham and Yarden 2011). Thus, our mouse cervix-derived single-cell colonies contain multiple lineages and can model cervix epithelial differentiation mediated by different signaling pathways. Wnt, along with Notch-inducing and EGFR pathways regulate stemness and differentiation of CVSCs.

### Three types of CVSC could be regulated by paracrine signaling pathways

**Fig. 6:**
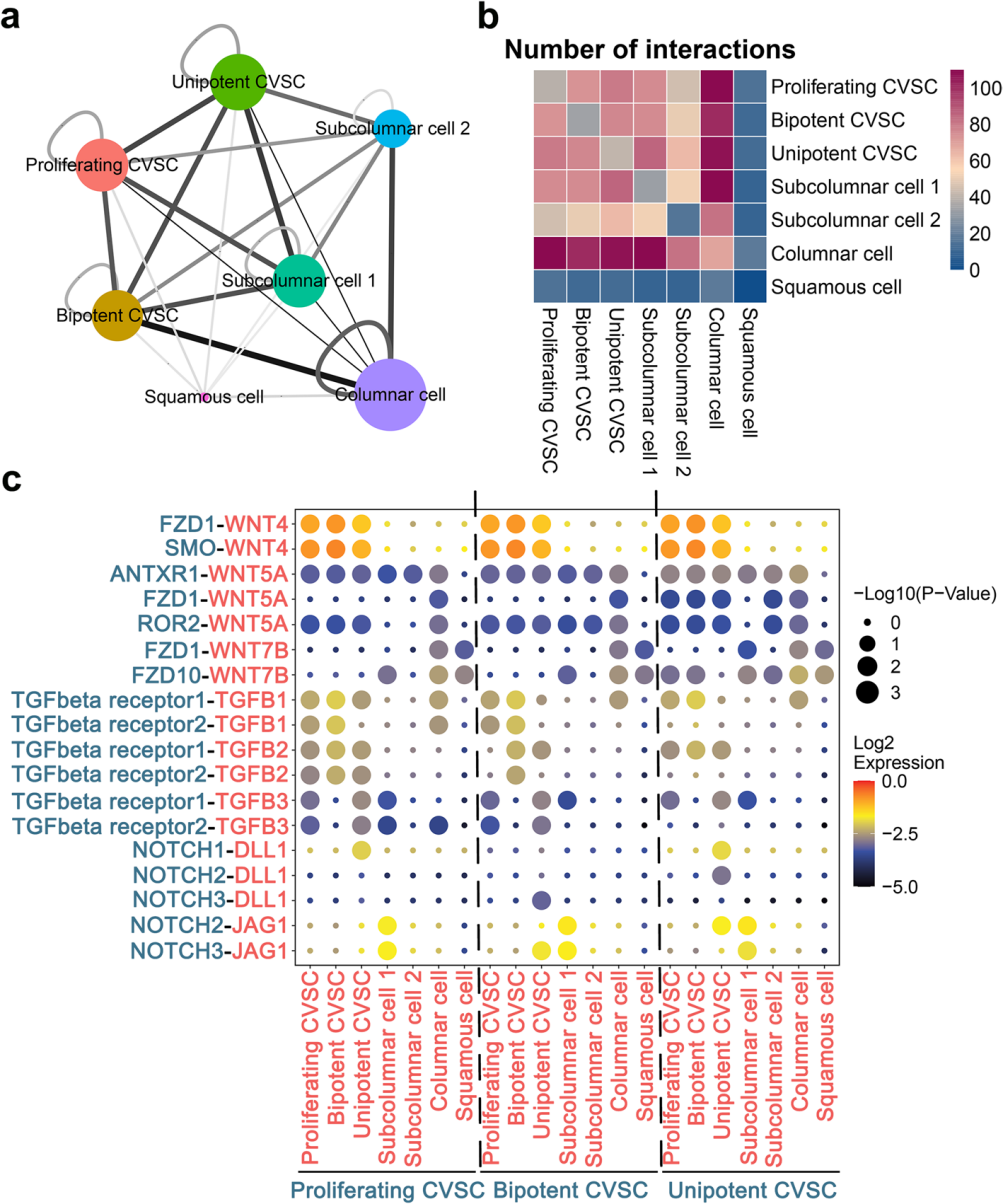
The interaction between CVSCs and other cells plays critical roles during cervical epithelium maturation. **a** Networks depicting cell types as nodes and interactions as edges. The size of the cell type is proportional to the total number of interactions of each cell type, and edge thickness is proportional to the number of interactions between the connecting types. The network layout was set to the force-directed layout. **b** Heatmap depicting the number of all possible interactions between the clusters analyzed. **c** Dot plot depicting selected cell-cell interactions enriched in cervical epithelium maturation

To further understand the relationship between different clusters of cells, especially their relationship with CVSCs, we inferred all possible intercellular communications by the expression of ligand-receptor pairs in both cell populations with CellPhoneDB. The result showed that the columnar cells had a remarkable interaction with the subcolumnar and three CVSC populations, which suggested that the paracrine effect between stem cells and differentiated secretory columnar cells is central to maintain the cervical epithelial homeostasis (Fig. 6a and b). We also analyzed the expression level of specific signaling pathway ligand-receptor pairs (Fig. 6c). Analysis of the predicted cell-cell interactions between three CVSCs and other cells revealed a wealth of signaling pathways including the Wnt, TGF-beta, and Notch pathways actively involved in the regulation process. For example, subcolumnar cells express relatively high levels of *Wnt7b*, the receptors of which *Fzd1* is expressed by unipotent CVSCs. This observation could explain our above conclusion that Wnt pathway play critical roles in CVSC self-renewal, and suggesting that maturing columnar cells function to provide Wnt ligand to anchor the CVSC at an undifferentiated status. In addition, columnar cells express relatively high levels of *Tgfb3*, the receptors of which (*TGFbeta receptor 2*) is expressed by proliferating CVSCs, which suggests a role for columnar cells in specifically regulating the cell cycle of proliferating CVSCs.

### Aberrant gene expression pattern in CVSCs is responsible for tumorigenesis

**Fig. 7:**
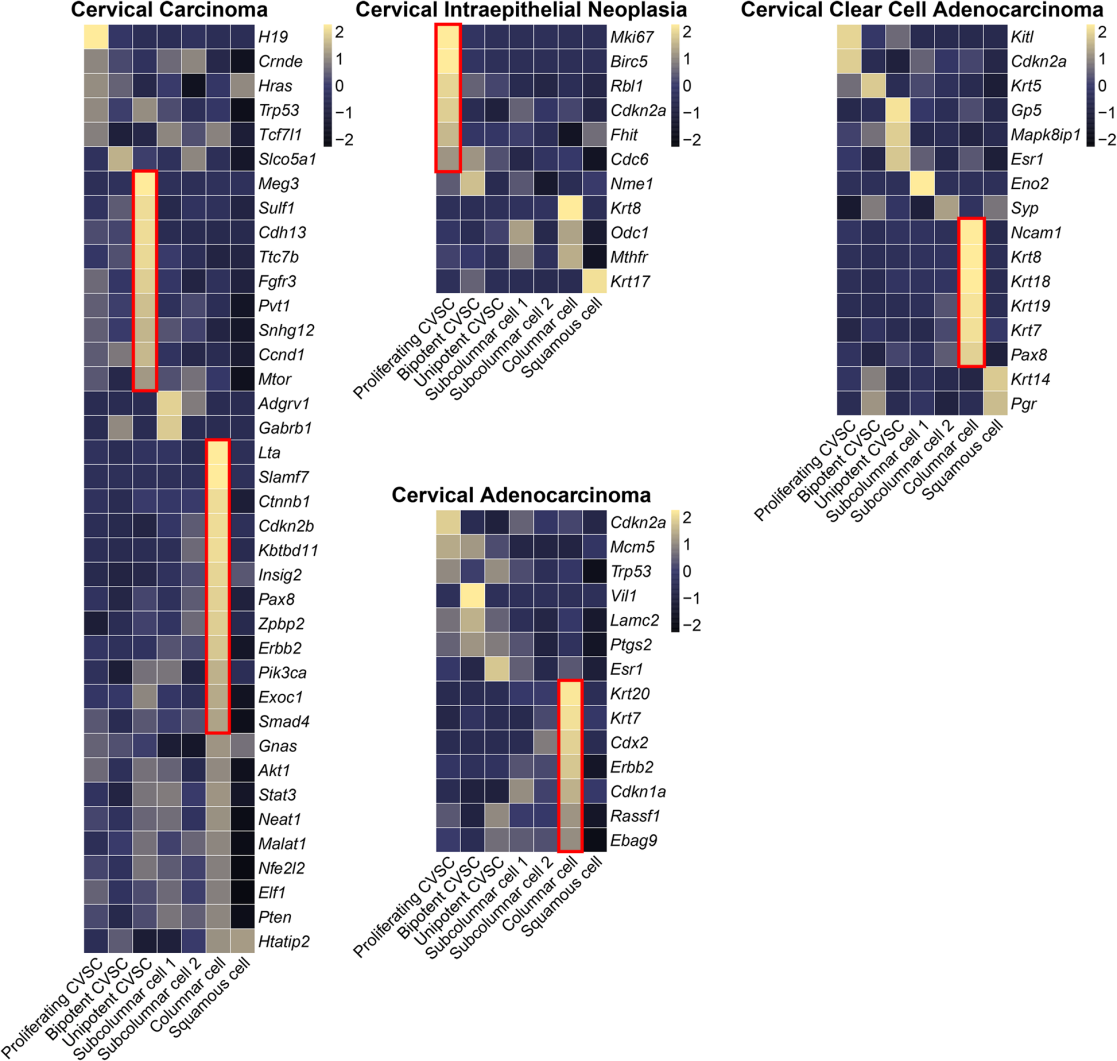
Putative tumor-initiating cells demonstrated by mapping of the cancer-associated genes. The average expression level of the human cervical cancer-related genes in each cluster. Mean expression values of the genes were calculated in each cluster. The color scheme is based on z-score distribution. Genes with maximum z-scores > 1 are shown in the red box. In the heatmap, each row represents one gene, and each column represents a single cell type (defined in Fig. 4)

The cellular origin of cervical diseases especially cervical tumors remains unclear, so here we utilized the single-cell transcriptomic data to dissect the cellular origin and underlying mechanism of such diseases. It was previously reported that certain types of cervical tumors were originated from stem cells (Schiffman et al. 2007; Martens et al. 2004). Here we explored whether the functions of specific cell types in the mouse cervix could be inferred from the expression pattern of genes whose aberrant function result in cervical disease (Fig. 7). We found that the mouse homologs of genes associated with cervical carcinoma were mostly expressed by unipotent CVSCs and columnar cells, confirming the major role of these cells in cervical carcinoma (Schiffman et al. 2007; Martens et al. 2004). Persistent genital infection with high-risk human papillomavirus is causally associated with cervical cancer and its precursors (cervical intraepithelial neoplasia, CIN) (Wiik et al. 2019). As another example, mouse homologs of genes that have been implicated in CIN, such as *Mki67*, *Birc5*, *Cdkn2a*, and *Rbl1*, were expressed specifically in the proliferating CVSCs, confirming the major role of these cells in CIN. Furthermore, since hyalinizing clear cell carcinoma (HCCC) is a rare minor salivary gland tumor made up of clear cells and forming cords and nests in a hyalinized stroma (Weinreb 2013), the expressions of genes associated with cervical clear cell adenocarcinoma, such as *Krt8*, *Krt18*, and *Krt19*, and *Krt7* showed strong enrichment for columnar cell-specific expression, confirming the major role of these cells in cervical clear cell adenocarcinoma. Thus, our single-cell transcriptomic analysis highlights specific cells responsible for tumor-related disorders.

## Discussion

Cervix is known to have huge regenerative potential, confirmed by the clinical observation that the cervical dimension can be restored to more than 90% of its original measurement within 6 months after conization (Song et al. 2016). Studying the principles of cellular regeneration and remodeling of the cervix is central to evaluate the impact of intrinsic and extrinsic epithelial disturbances, as well as for prospective and therapeutic strategies for cervical disease. Although the repair and regeneration of the cervical epithelium in response to damage caused by excessive infection, inflammation, or trauma is well recognized (Phadnis et al. 2011; Geirsson et al. 1977), the cell types that contribute to the replenishment of the damaged tissue remain poorly understood.

In the present study, we established an in vitro feeder-based culture system that allows selective cloning, large-scale proliferative expansion of a stem cell population derived from human and mouse cervix epithelium. Here we provide the first glimpse into the characteristics of the putative CVSCs and reveal their bi-lineage differentiation potential to give rise to either columnar or squamous cells. This cervical stem cell population marked by KRT5 and P63 expression with properties of self-renewal was identified in both mice and humans. As shown by the lineage tracing followed by TCA injury, reserve KRT5 + P63+ cells rapidly respond to injury within a few days. They can initiate epithelial regeneration through differentiation as they migrate towards the cervical luminal surface. This differentiation pattern was further observed in the in vitro 3D Matrigel differentiation assay. In the self-organized CVSCs spheroids, the renewing P63+ cells are located at the outer layer of the sphere, representing the basement membrane of the epithelial luminal, while the differentiated cells expressing columnar or squamous makers lie inside the luminal space, showing a histological structure that resembles the cervix cavity. The spatial differentiation pattern indicates that there is an obvious relation between the activity of the CVSCs and the histological structure of the tissue, which is a universality shared by multiple epithelial tissues, especially for those showing rapid cell turnover (Cliffe et al. 2005; Slack 2000).

Adult epithelial stem cells are considered to exist in most, if not all, epithelial tissues and organs, which are capable of repopulating the original cell population and re-building the epithelial barrier. These tissue-specific stem/progenitor populations can form the differentiated cells of their particular tissue type but not those of any other. For the cervix, the epithelium consists of ectocervical squamous epithelium that includes a basal cell layer, endocervical columnar epithelium, and subcolumnar reserve cells, so there are several potential candidates to qualify as cervical stem cells. It is already known that subcolumnar reserve cells are undifferentiated, omnipotent cells that possess the capacity to undergo metaplasia in the TZ (Voojis et al. 2008). The cervical basal cells that exist under ectocervical squamous epithelium are more differentiated, dedicated to the formation of squamous cells, and therefore less suitable as stem cells of the epithelium (Smedts et al. 1993). Another unsolved issue of identification of the cervical stem cell is that, so far, reliable markers are not available to identify stem cells of certain epithelia. To overcome these challenges, we have sequenced single cells from 10 donor mice using 10X genomics single-cell RNA sequencing. The existence of a mouse cervix stem/progenitor population and its active cell type was identified. Pseudo-time reconstitution identified bipotent and unipotent subtypes, and indicated that the bipotent sub-population can give rise to either squamous epithelial cell fates or columnar epithelial cell fates mediated by different signaling pathways. This differentiation trajectory was further confirmed by a real-time reconstitution achieved by bulk RNA sequencing of 3D cultures at different differentiation time points. Notably, the differentiation path of two single cell clones exhibited lineage plasticity in the correlation heatmap, evidenced by the finding that the proliferating, unipotent, and bipotent CVSCs can convert to each other. This intriguing finding raised the question that whether this cellular heterogeneity in CVSC population reflects the existence of distinct progenitors or, alternatively, a single multipotent progenitor with remarkable plasticity. Further studies can be focused on a more intensive evaluation of the single cell-derived clones and their cell fate dynamics in normal and pathological conditions.

The current data also provide insight into cervix epithelial regeneration, as well as disease pathogenesis. It is demonstrated that cervical carcinoma is tightly associated with the dysfunction of unipotent CVSCs and columnar epithelial clusters.

Altogether, this study aims to report the establishment of a self-renewed, clonogenic cell population derived from mouse or human cervical epithelium, as well as to provide detailed information about the cytology of the mouse cervix. To this end, we isolated, expanded, and fully characterized the cloned cells. Their differentiation potential was carefully analyzed by lineage tracing and 3D differentiation in vivo and in vitro. Furthermore, single-cell transcriptomics was used to comprehensively resolve the epithelial cell atlas that is involved in cervical cell turnover. The obtained dataset provides an in-depth view of cell types in the mouse cervix at single-cell resolution, enabling comparison of gene expression patterns among cell types, as well as the exploration of the gene expression profiles related to diseases, cell-type-specific ligand-receptor interactions, and bi-lineage differentiation mediated by different signaling pathways. Our research on adult cervix epithelium and the CVSCs contributes to our understanding of endogenous cervix repair mechanisms.

## Supplementary Information

**Additional file 1: Figure S1.** Validation of marker gene expression in murine and human CVSCs. a Putative mouse CVSC colonies co-stained with cervical epithelial stem cell markers KRT5 and P63. Data representative of *n* = 10 biological replicates. Scale bar, 100 μm. b Putative human CVSC colonies stained with proliferative marker KI67, adult epithelial stem cell marker P63, cervical squamous cell marker KRT10, and cervical columnar cell marker MUC1. Data representative of *n* = 3 biological replicates. Scale bar, 100 μm. c qPCR analysis showing the level of stem/progenitor cell marker (P63, KRT5, STMN1 and NRTN) gene expression of mouse cervix sample and mCVSC. *n* = 3, biological replicates. Error bars, SD. d qPCR showing squamous (SPRR1B and KRT10) and columnar (KRT8 and KRT18) epithelium marker gene expression of mouse cervix sample and mCVSC. *n* = 3, biological replicates. Error bars, SD.

**Additional file 2: Figure S2.** Lineage tracing experiments in Krt5 Cre^ERT2^-Gt(ROSA)26Sor^tm4(ACTB-tdTomato-EGFP)^ mice. a Lineage tracing experiments in Krt5 Cre^ERT2^-Gt(ROSA)26Sor^tm4(ACTB-tdTomato-EGFP)^ mice treated with low-dose tamoxifen to label only a few single KRT5+ cells and traced their fate 10 days post labeling (Sham). Data representative of *n* = 5 biological replicates. Scale bar, 100 μm. b Lineage tracing experiments in Krt5 Cre^ERT2^-Gt(ROSA)26Sor^tm4(ACTB-tdTomato-EGFP)^ mice treated with low-dose tamoxifen prior to TCA injury reveals that squamous metaplasia arising after the injury is positive for Krt5-GFP lineage marker on Day10. Data representative of *n* = 5 biological replicates. Scale bar, 100 μm.

**Additional file 3: Figure S3.** Quality control of single-cell RNA-seq. a Histograms show the distribution of the cells from single-cell RNA-seq ordered by the number of detected genes and mitochondrial gene expression content passed the quality control. b Total numbers of cells that passed the quality control, processed by single-cell RNA-seq. Each row is a separate cluster. c The original UMAP presentation of major cell types in cervical epithelial cells without removing immune cells. d The gene expression levels of immune cell marker in original cervical epithelial cells. Cluster 8 (*Ptprc*+) was excluded in further analysis.

**Additional file 4: Figure S4.** Two types of CVSC represent different biological lineages in the cervix. a Left, cell trajectory map of cervical epithelial cells showing the pseudo-time; Right, pseudo-time trajectory analysis shows the putative differentiation paths from CVSC to columnar cells or squamous cells. b The pseudo-time trajectory showing the distribution of CVSC. c Immunostaining patterns for KRT17 and P63 of the endocervical columnar cells. The green arrow indicates P63+/KRT17- CVSC population and the red arrow indicates P63+/KRT17+ CVSC population. Data representative of *n* = 3 biological replicates. Scale bar, 100 μm. d Representative GO terms and pathways enriched in cluster-specific marker genes based on functional enrichment analysis (*p* < 0.01).

**Additional file 5: Figure S5.** Wnt, Notch and EGFR signaling pathways play a role in cervical epithelium differentiation. a Expression of specific genes along the cell trajectory important for the corresponding differentiation paths. The dots indicate the gene expression of individual cells colored by the cervical epithelial cell type. The black lines approximate expression along the inferred trajectory by polynomial regression fits. b Upper: Gene Set Enrichment Analysis is performed using the Hallmark Wnt Gene Set with differentially expressed genes between A_Day0 and A_Day6. Lower: Gene Set Enrichment Analysis is performed using the Hallmark EGFR Gene Set with differentially expressed genes between B_Day0 and B_Day4. c Expression analysis of downstream of EGFR target genes in mouse CVSC single-cell colonies at different days after aggregation and culture.

**Additional file 6: Table 1.** qPCR primer information

## Data Availability

The datasets used and/or analysed during the current study are available from the corresponding author on reasonable request.

## Abbreviations

CVSCs: Cervical stem/progenitor cells
SPF: Specific pathogen Free
SOP: Standard Operating Procedure
CFE: Colony forming efficiency
TCA: Trichloroacetic acid
FPKM: FragmentsPer Kilobase per Million
GO: Gene Ontology
TZ: Transformation zone
UMAP: Uniform manifold approximation and projection
CIN: Cervical intraepithelial neoplasia
HCCC: Hyalinizing clear cell carcinoma
GSEA: Gene set enrichment analysis
