# Supplementary figures and images for: Single-cell analysis defines the lineage plasticity of stem cells in cervix epithelium
Source: Cell Regen. 2021 Nov 1;10:36. doi: 10.1186/s13619-021-00096-2 (PMC8558147; doi:10.1186/s13619-021-00096-2)

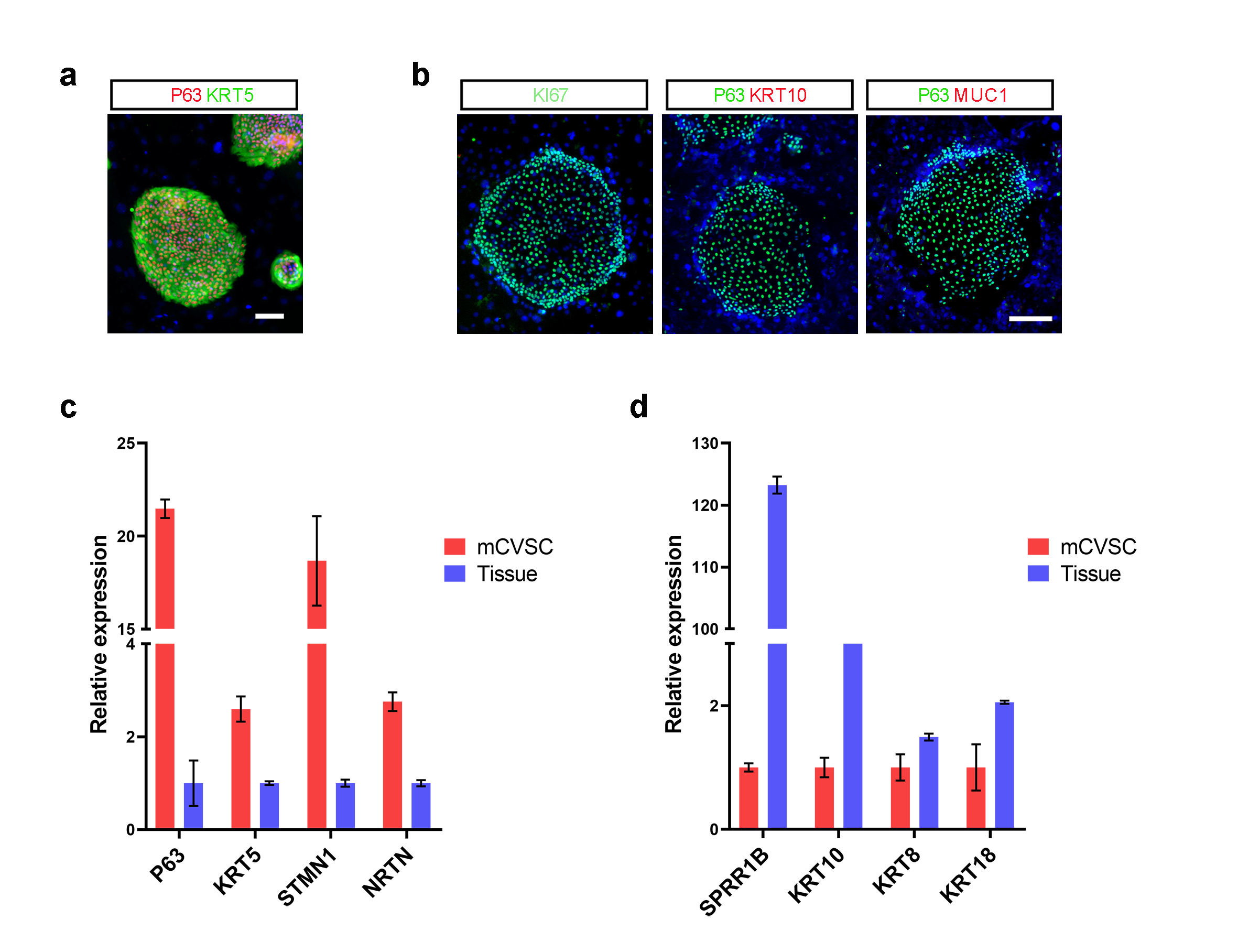

Supplement: Supplementary file 1 — Additional file 1: Figure S1. Validation of marker gene expression in murine and human CVSCs. a Putative mouse CVSC colonies co-stained with cervical epithelial stem cell markers KRT5 and P63. Data representative of n = 10 biological replicates. Scale bar, 100 μm. b Putative human CVSC colonies stained with proliferative marker KI67, adult epithelial stem cell marker P63, cervical squamous cell marker KRT10, and cervical columnar cell marker MUC1. Data representative of n = 3 biological replicates. Scale bar, 100 μm. c qPCR analysis showing the level of stem/progenitor cell marker (P63, KRT5, STMN1 and NRTN) gene expression of mouse cervix sample and mCVSC. n = 3, biological replicates. Error bars, SD. d qPCR showing squamous (SPRR1B and KRT10) and columnar (KRT8 and KRT18) epithelium marker gene expression of mouse cervix sample and mCVSC. n = 3, biological replicates. Error bars, SD. [file 13619_2021_96_MOESM1_ESM.tif]

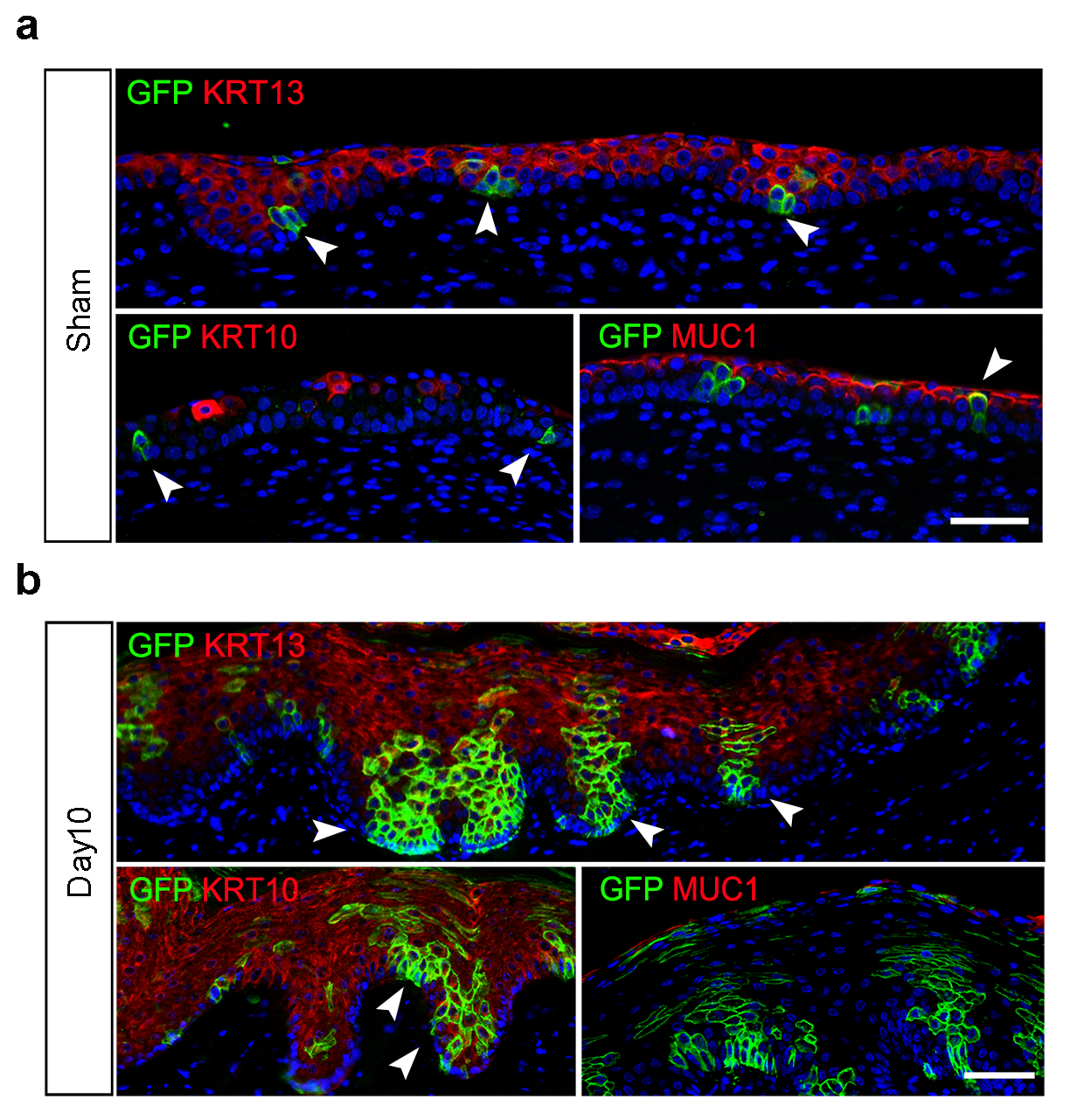

Supplement: Supplementary file 2 — Additional file 2: Figure S2. Lineage tracing experiments in Krt5 CreERT2-Gt(ROSA)26Sortm4(ACTB-tdTomato-EGFP) mice. a Lineage tracing experiments in Krt5 CreERT2-Gt(ROSA)26Sortm4(ACTB-tdTomato-EGFP) mice treated with low-dose tamoxifen to label only a few single KRT5+ cells and traced their fate 10 days post labeling (Sham). Data representative of n = 5 biological replicates. Scale bar, 100 μm. b Lineage tracing experiments in Krt5 CreERT2-Gt(ROSA)26Sortm4(ACTB-tdTomato-EGFP) mice treated with low-dose tamoxifen prior to TCA injury reveals that squamous metaplasia arising after the injury is positive for Krt5-GFP lineage marker on Day10. Data representative of n = 5 biological replicates. Scale bar, 100 μm. [file 13619_2021_96_MOESM2_ESM.tif]

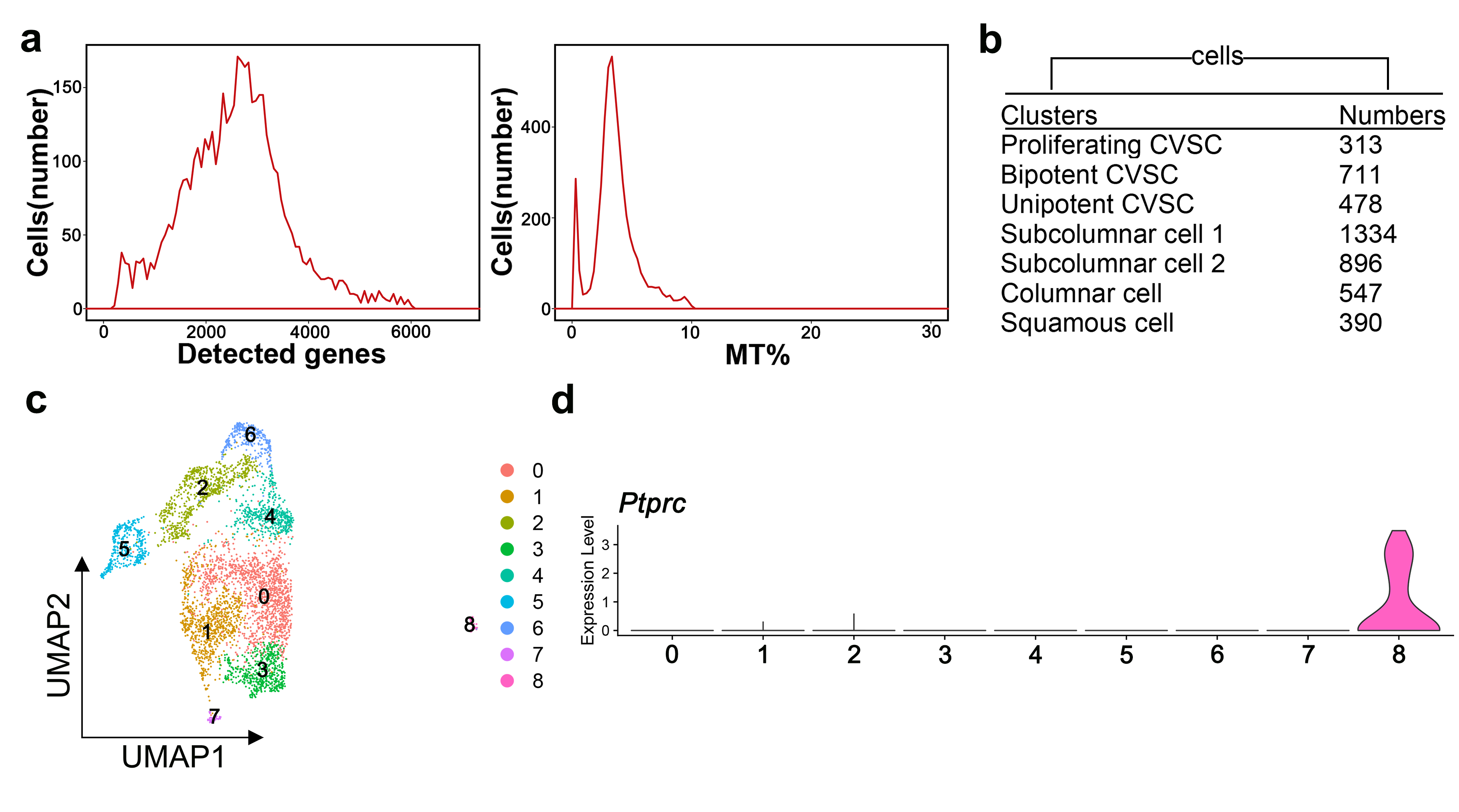

Supplement: Supplementary file 3 — Additional file 3: Figure S3. Quality control of single-cell RNA-seq. a Histograms show the distribution of the cells from single-cell RNA-seq ordered by the number of detected genes and mitochondrial gene expression content passed the quality control. b Total numbers of cells that passed the quality control, processed by single-cell RNA-seq. Each row is a separate cluster. c The original UMAP presentation of major cell types in cervical epithelial cells without removing immune cells. d The gene expression levels of immune cell marker in original cervical epithelial cells. Cluster 8 (Ptprc+) was excluded in further analysis. [file 13619_2021_96_MOESM3_ESM.tif]

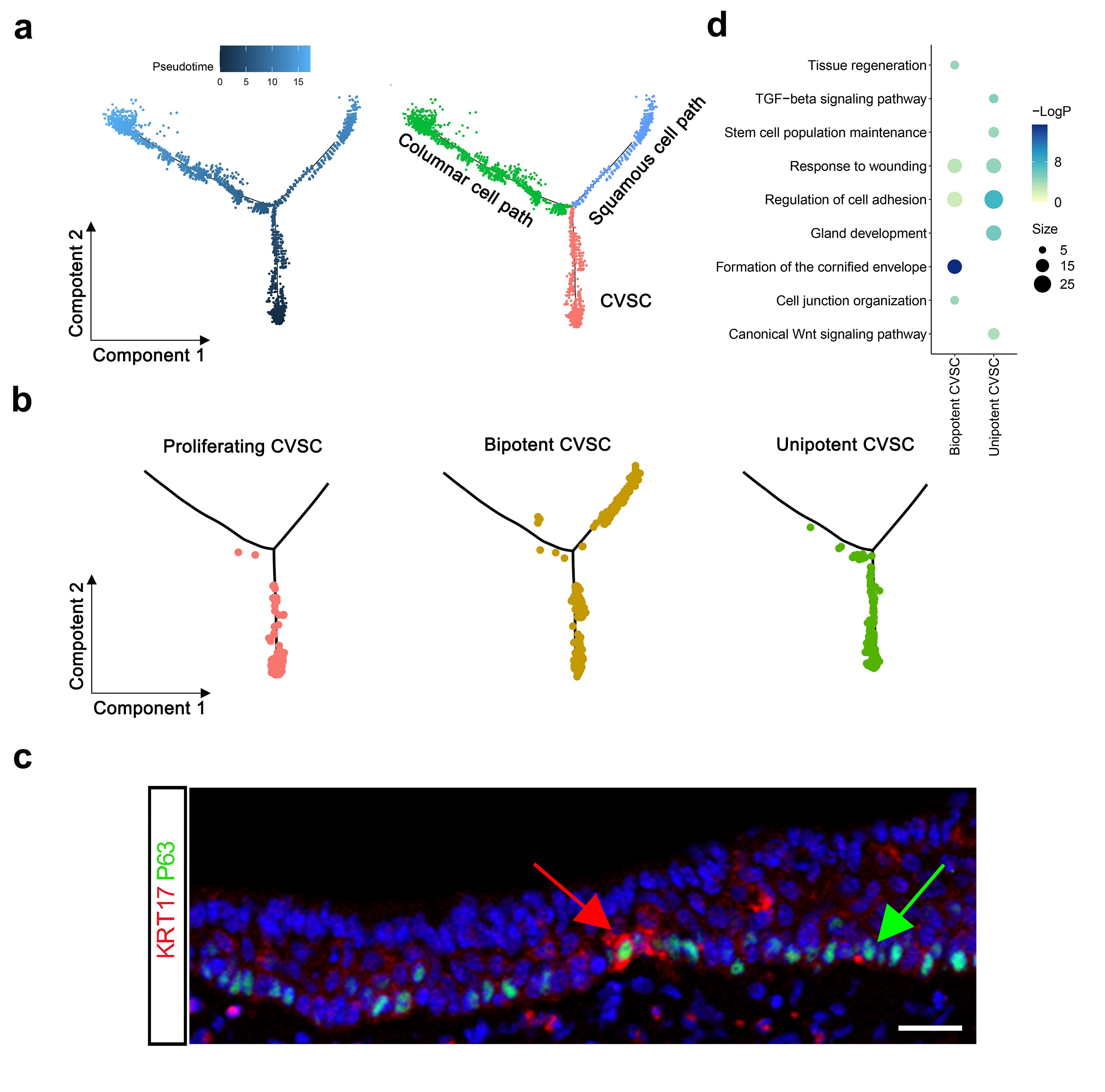

Supplement: Supplementary file 4 — Additional file 4: Figure S4. Two types of CVSC represent different biological lineages in the cervix. a Left, cell trajectory map of cervical epithelial cells showing the pseudo-time; Right, pseudo-time trajectory analysis shows the putative differentiation paths from CVSC to columnar cells or squamous cells. b The pseudo-time trajectory showing the distribution of CVSC. c Immunostaining patterns for KRT17 and P63 of the endocervical columnar cells. The green arrow indicates P63+/KRT17- CVSC population and the red arrow indicates P63+/KRT17+ CVSC population. Data representative of n = 3 biological replicates. Scale bar, 100 μm. d Representative GO terms and pathways enriched in cluster-specific marker genes based on functional enrichment analysis (p < 0.01). [file 13619_2021_96_MOESM4_ESM.tif]

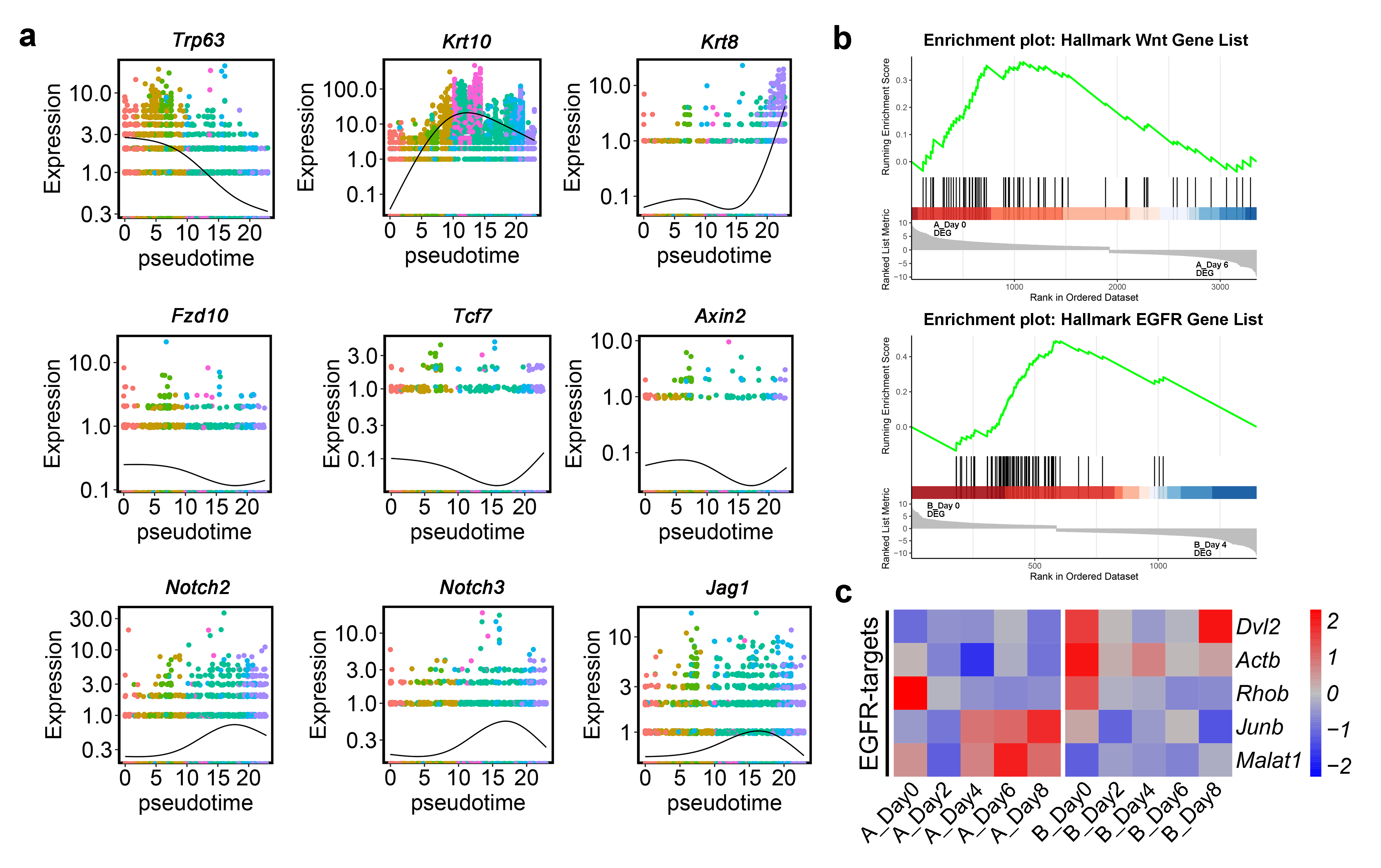

Supplement: Supplementary file 5 — Additional file 5: Figure S5. Wnt, Notch and EGFR signaling pathways play a role in cervical epithelium differentiation. a Expression of specific genes along the cell trajectory important for the corresponding differentiation paths. The dots indicate the gene expression of individual cells colored by the cervical epithelial cell type. The black lines approximate expression along the inferred trajectory by polynomial regression fits. b Upper: Gene Set Enrichment Analysis is performed using the Hallmark Wnt Gene Set with differentially expressed genes between A_Day0 and A_Day6. Lower: Gene Set Enrichment Analysis is performed using the Hallmark EGFR Gene Set with differentially expressed genes between B_Day0 and B_Day4. c Expression analysis of downstream of EGFR target genes in mouse CVSC single-cell colonies at different days after aggregation and culture. [file 13619_2021_96_MOESM5_ESM.tif]
